# TLR2 and TLR4 in Parkinson’s disease pathogenesis: the environment takes a toll on the gut

**DOI:** 10.1186/s40035-021-00271-0

**Published:** 2021-11-17

**Authors:** Anastazja M. Gorecki, Chidozie C. Anyaegbu, Ryan S. Anderton

**Affiliations:** 1 School of Biological Science, University of Western Australia, Crawley, WA Australia; 2 Neurodegenerative Disorders Research Group, Perron Institute for Neurological and Translational Science, Nedlands, WA Australia; 3 Curtin Health Innovation Research Institute, Ralph and Patricia Sarich Neuroscience Research Institute, Curtin University, Nedlands, WA Australia; 4 Faculty of Medicine, Nursing and Midwifery and Faculty of Health Sciences, University of Notre Dame Australia, Fremantle, WA Australia; 5 School of Nursing, Midwifery, Health Sciences and Physiotherapy, University of Notre Dame Australia, Fremantle, WA Australia

**Keywords:** Parkinson’s disease, TLR2, TLR4, α-Synuclein, Gut-brain axis, Gut barrier, Gut homeostasis

## Abstract

Parkinson’s disease (PD) is an incurable, devastating disorder that is characterized by pathological protein aggregation and neurodegeneration in the substantia nigra. In recent years, growing evidence has implicated the gut environment and the gut-brain axis in the pathogenesis and progression of PD, especially in a subset of people who exhibit prodromal gastrointestinal dysfunction. Specifically, perturbations of gut homeostasis are hypothesized to contribute to α-synuclein aggregation in enteric neurons, which may spread to the brain over decades and eventually result in the characteristic central nervous system manifestations of PD, including neurodegeneration and motor impairments. However, the mechanisms linking gut disturbances and α-synuclein aggregation are still unclear. A plethora of research indicates that toll-like receptors (TLRs), especially TLR2 and TLR4, are critical mediators of gut homeostasis. Alongside their established role in innate immunity throughout the body, studies are increasingly demonstrating that TLR2 and TLR4 signalling shapes the development and function of the gut and the enteric nervous system. Notably, TLR2 and TLR4 are dysregulated in patients with PD, and may thus be central to early gut dysfunction in PD. To better understand the putative contribution of intestinal TLR2 and TLR4 dysfunction to early α-synuclein aggregation and PD, we critically discuss the role of TLR2 and TLR4 in normal gut function as well as evidence for altered TLR2 and TLR4 signalling in PD, by reviewing clinical, animal model and in vitro research. Growing evidence on the immunological aetiology of α-synuclein aggregation is also discussed, with a focus on the interactions of α-synuclein with TLR2 and TLR4. We propose a conceptual model of PD pathogenesis in which microbial dysbiosis alters the permeability of the intestinal barrier as well as TLR2 and TLR4 signalling, ultimately leading to a positive feedback loop of chronic gut dysfunction promoting α-synuclein aggregation in enteric and vagal neurons. In turn, α-synuclein aggregates may then migrate to the brain via peripheral nerves, such as the vagal nerve, to contribute to neuroinflammation and neurodegeneration typically associated with PD.

## Background

Parkinson’s disease (PD) is a debilitating and incurable neurodegenerative disorder, principally characterised by intraneuronal accumulation of α-synuclein aggregates called Lewy bodies, neurodegeneration in the nigrostriatal pathway and motor impairments. For the majority of PD cases, there is no known genetic cause, and these idiopathic cases exhibit greater clinical and pathological heterogeneity than familial forms of the disease. Idiopathic PD presents as a multi-systemic disorder, wherein a plethora of genetic, environmental, and molecular factors are thought to contribute to diverse disease presentation, pathology, and progression [1, 2]. The past decade has witnessed a wealth of evidence implicating the gut in whole-body health, as well as the pathogenesis of various diseases including PD. Importantly, an altered gut microbiome and a pro-inflammatory gut environment are thought to contribute to the gut-to-brain spread of α-synuclein and thus may be central to Lewy body-mediated neurodegeneration. However, the link between an altered gut environment and α-synuclein pathology is still unclear. The gut barrier exists in a precarious state of homeostasis, governed by commensal gut microbes, epithelial cells, the immune system, and exogenous factors present in the lumen. The intestinal epithelial cells and the underlying immune cells express receptors for pathogen-associated molecular patterns (PAMPs), such as toll-like receptors (TLRs), with which they detect microbial components. In addition to their role in innate immunity and host-microbe signalling, TLRs in epithelial cells and enteric neurons also mediate gut function, so dysregulated TLR signalling can exacerbate microbial dysbiosis and inflammation [3]. Growing research indicates that the TLR-mediated immune responses contribute to PD progression, due to the chronic activation of gut TLRs from microbial dysbiosis [4] or α-synuclein-induced neuroinflammation in the central nervous system (CNS) [5]. Specifically, TLR2 and TLR4 are repeatedly implicated in PD, with reported changes in receptor levels or signalling in people with PD, a central role in neurodegeneration in animal models, and molecular interactions with α-synuclein in vitro. In this review, we will focus on these two receptors, and discuss compelling evidence for an early and direct role of TLR2 and TLR4 in enteric α-synuclein aggregation in PD. Our review synthesizes recent evidence concerning the role of TLR2 and TLR4 in gut homeostasis and the enteric nervous system (ENS) function in light of altered TLR2 or TLR4 signalling and gut dysfunction in PD. We propose that TLR2 and TLR4 signalling may contribute to PD pathogenesis by increasing gut permeability and inflammation, which can drive α-synuclein aggregation in the gut or brain.

## The gastrointestinal tract in PD

Once viewed as a simple conduit for mechanical and chemical digestion, the gastrointestinal tract is increasingly recognised as a complex system which influences overall health. The gastrointestinal tract encompasses a series of digestive organs composed of the epithelial barrier, endocrine cells, muscular layers, the second largest neuronal population (the ENS) and the body’s principal collection of immune cells. A complex and dynamic ecosystem of bacteria, protozoa, and fungi, termed the gut or intestinal microbiota, resides symbiotically within the gut lumen and along the gut mucosa, endowing the host with many critical functions including metabolism of otherwise-inaccessible dietary compounds, protection from pathogens and patterning of the immune system [6]. As such, the intestinal barrier is one of the first and largest interaction sites between host biology and external factors, facilitating the absorption of nutrients while preventing the entry of luminal microbes, toxins, and other gut contents. The overall function of the gut is regulated by complex interactions between luminal contents, gut microbes, epithelial cells, enteroendocrine cells and immune cells, as well as the ENS and CNS [7]. Changes to the gut environment, such as those arising from dietary components or toxin exposure, can shift the microbiota towards a pro-inflammatory or pathogenic community (termed microbial dysbiosis), increase the permeability of the epithelial barrier (‘leaky gut’) and trigger gut inflammation. In turn, such gut dysfunction can have significant downstream consequences on the body, and is linked with the development or exacerbation of various diseases.

Various lines of evidence are converging to implicate chronic gut dysfunction and immune activation as triggers and drivers of PD pathology. People with PD exhibit many features reflecting inflammatory bowel disease (IBD) (reviewed in [8]), including altered gut microbiome composition and function [9–13], a leaky gut [14–16], widespread immune activation (reviewed in [17]) and a broad spectrum of gastrointestinal symptoms (e.g., constipation, nausea, bloating) [18, 19], some of which may appear decades prior to PD diagnosis [20]. Likewise, various genetic (such as *LRRK2* and *TNF-α* variants) [21, 22], environmental (e.g., pesticides) or lifestyle factors (diet, exercise, or antibiotic use) [23, 24] which mediate PD risk or progression, are also linked with intestinal inflammation and IBD, while IBD is itself known to be a risk factor for PD [25, 26].

Idiopathic PD is characterised by the misfolding and aggregation of α-synuclein into Lewy bodies and Lewy neurites with associated nigrostriatal dopaminergic neuronal death, but the triggers and factors leading to α-synuclein pathology are not fully understood. Gut dysfunction has been hypothesised to contribute to pathogenic α-synuclein aggregation throughout the ENS, which may then spread to the brain. Evidence of propagation of α-synuclein pathology from the ENS to the brain via the vagus nerve has been found in various rodent models of PD [27–30], while the incidence of PD is lower among people with a history of vagotomy [31], demonstrating the importance of gut to brain propagation of α-synuclein. The systematic neuropathological studies of PD brains by Braak (2003) suggest that, upon reaching the dorsal motor nucleus of the vagus nerve in the medulla oblongata from a peripheral structure, α-synuclein aggregation and Lewy body formation continue to ascend to the upper brain stem, leading to nigrostriatal neurodegeneration (the point of motor symptom onset) and subsequent spread and neurodegeneration throughout the cerebrum, with six hypothesised ‘Braak’ stages of degeneration referred to as ‘Braak staging’ [32, 33]. However, more recent studies support a heterogenous pattern and rate of neuropathology. For example, conflicting evidence exists concerning vagotomies and PD risk among people [31, 34], and Braak staging is suggested to apply only to early-onset, long-lasting PD but not late-onset and rapidly progressing cases [35], which may also exhibit other age-associated neurodegenerative features such as amyloid beta (Aβ) and tau pathologies. A recent study using multimodal imaging of people with PD has addressed such controversy, reporting two discrete types of PD with either ‘brain-first’ (CNS) or ‘body-first’ (peripheral nervous system or ENS) trajectory [36], a model which is proposed to account for varied α-synuclein pathology, motor and non-motor symptom heterogeneity, and differing rates of disease progression. Coupled with the complex nature of PD in regard to genetic predisposition, environmental risk factors, cellular mechanisms and clinical presentation, such disease sub-typing may elucidate a clearer understanding of PD pathogenesis, pathology, and eventual therapies.

Despite evidence for a role of gut inflammation and gut-brain α-synuclein spread in PD, the origin of pathogenic intestinal α-synuclein aggregation in the gut is still unclear. However, evidence implicating gut-related TLR2 and TLR4 signalling in the pathogenesis of ‘body-first’ PD pathology is growing, in light of: 1) altered expression and signalling of both TLR2 and TLR4 in people with PD, especially in the gastrointestinal system; 2) the role of TLR2 and TLR4 in gut homeostasis and thus leaky gut and inflammation; and 3) the proposed role of α-synuclein as an immune signalling molecule and its ability to activate TLR2 and TLR4.

## TLRs

Found on both immune and non-immune cell populations throughout the body, TLRs are transmembrane proteins which recognise PAMPs and cellular damage-associated molecular patterns (DAMPs) to initiate innate and adaptive immune responses. In terms of PAMP recognition, cell surface TLRs predominately respond to bacterial ligands (TLRs 1, 2, 4, 5, 6 and 10) while intracellular TLRs localised to the endoplasmic reticulum, endosomes and lysosomes recognise bacterial and viral nucleic acids (TLRs 3, 7, 8, 9, 11, 12 and 13) (reviewed in [37]). DAMPs are various molecules released into the extracellular matrix from stressed, injured or dying cells which can activate TLRs to regulate inflammatory genes and tissue repair [38]. Activation of TLRs by PAMPs and DAMPs is crucial for pathogen defence and tissue repair throughout the body; however, dysfunctional or excessive TLR activation and downstream signalling can contribute to various autoimmune, inflammatory, and age-associated diseases in various body systems, such as PD [4, 5, 39].

### TLR2

TLR2 is a cell-surface receptor expressed on various immune and epithelial cells, as well as neuronal and glial cells in the central, peripheral and enteric nervous systems [40]. TLR2 forms homodimers or heterodimers with TLR1 and TLR6 to recognise a variety of gram-negative and gram-positive bacterial products such as lipoteichoic acid, lipoproteins, peptidoglycans and bacterial amyloids (e.g., curli protein) [41], as well as endogenous factors including α-synuclein, the latter of which will be discussed in detail below. TLR2 activation may lead to pro-inflammatory and anti-inflammatory responses depending on the cell type on which it is expressed, the ligand and the co-receptor (reviewed in [42]). For example, on dendritic cells, TLR2 activation by peptidoglycans, but not lipoteichoic acid, induces tumour necrosis factor-α (TNF-α) release. Interestingly, activation of TLR2 by Aβ42 aggregates in microglial cells causes pro-inflammatory cytokine release, and this response is enhanced by TLR1 co-receptor action but suppressed by TLR2-TLR6 activation [42]. Conversely, TLR2 activation by polysaccharide A from *Bacteroides fragilis* causes an anti-inflammatory response in B cells with interleukin 10 (IL-10) production [43], indicating a complex immunoregulatory role of TLR2 in response to various pathogens that are often present in the gut or endogenous factors that are implicated in neurodegeneration.

#### TLR2 expression is altered in people with PD

In light of an established role in innate immunity, TLR2 likely plays a crucial part in microbial-induced inflammation throughout the whole body, and several lines of evidence demonstrate alterations of TLR2 signalling in people with PD. First, a C-to-T single nucleotide polymorphism in *TLR2* rs3804099  is associated with increased PD risk in a Han Chinese population, especially among those with late-onset PD [44], whereas a recent study utilising a Caucasian subset of the Parkinson’s Progression Markers Initiative (PPMI) cohort reported that TC heterozygotes and minor CC homozygotes of *TLR2* rs3804099 have  significantly increased PD risk [45], indicating possible geographical or ethnic differences regulating the link between *TLR2* polymorphisms and PD. This polymorphism is a significant expression quantitative trait loci (eQTL) in certain cell types, which is predicted to influence *TLR2* mRNA levels [45] and is associated with susceptibility to bacterial infections [46, 47], however further research is needed to confirm the direct effect of *TLR2* rs3804099 on protein levels.

TLR2 expression is increased in circulating monocytes of people with PD [48, 49]. Post-mortem analysis of brain samples from people with PD has demonstrated increased neuronal and microglial TLR2 expression in various brain regions, including the substantia nigra and putamen [50], and increased nigrostriatal expression of TLR2-associated signalling pathways (including cluster of differentiation 14 (CD14), the co-receptor for TLR4) [51]. This is particularly striking considering the very low expression of TLR2 in post-mortem healthy brains, which is concentrated in microglia [52]. Accordingly, it has been suggested that the elevated levels of neuronal TLR2 in people with PD is a result of disease processes [5]. Interestingly, TLR2 expression in the substantia nigra of people with PD is significantly higher than that of healthy controls, but significantly lower than that in people with incidental Lewy Body disease [53] (a prodromal, asymptomatic stage of PD), thus supporting the role of TLR2 in early disease processes. Furthermore, this study reported an association between nigrostriatal TLR2 expression and primed microglia in incidental Lewy Body disease, leading the authors to propose that the upregulation of TLR2 may represent early microglial activation prior to extensive PD pathology and neurodegeneration and that such early microglial activation may arise due to an interaction between TLR2 and oligomeric α-synuclein [53].

### TLR4

TLR4 is widely expressed on the surface of many cells throughout the human body, including, but not limited to, immune cells, intestinal cells, microglia, astrocytes and neurons [54–56]. Mostly known for its ability to bind lipopolysaccharides (LPS) from gram-negative bacteria, recent studies indicate that TLR4 also interacts with other PAMPs and DAMPs, including PD-associated α-synuclein [52, 57, 58]. Upon ligand binding, TLR4 activates the signalling of nuclear factor kappa-light-chain-enhancer of activated B cells (NF-κB) and the downstream production of pro-inflammatory cytokine, playing an important role in innate immune defence, protein clearance mechanisms (e.g., autophagy and glial phagocytosis), as well as microglial activation, neuronal excitotoxicity and neurodegeneration [39]. For LPS-TLR4 signalling, LPS-binding protein (LBP) attaches LPS and presents it to CD14, which together with myeloid differentiation factor-2 protein (MD-2), alters TLR4 localisation on the cellular membrane to facilitate TLR4 activation. This in turn leads to the activation of signalling pathways involving both myeloid differentiation primary response protein 88 (MyD88)-dependent and MyD88-independent signals, both leading to NF-κB activation and pro-inflammatory cytokine release (summarised in [59]).

#### TLR4 levels are altered in PD

Reflecting the inflammatory nature of the disease, a growing number of studies support a role for altered TLR4 signalling in PD pathology. First, people with PD exhibit increased levels of TLR4 protein in colonic biopsies, circulating monocytes and post-mortem caudate and putamen samples [48, 60]. Studies have also demonstrated perturbed serum markers indicative of TLR4 activation (as assessed through altered LPS-binding protein levels) [14] and elevated endogenous DAMP high-mobility group box protein 1 (HMGB1) levels [61] in people with PD compared to healthy controls. The HMGB1–TLR4 axis induces secretion of various pro-inflammatory cytokines, and expression levels of both TLR4 and HMGB1 are positively associated with PD progression and disease duration, and negatively associated with the effectiveness of pharmacological treatment [61]. Interestingly, in healthy people, the substantia nigra and putamen exhibit the highest level of TLR4 expression among all human brain structures [52], indicating that unchecked TLR4 expression and signalling are not solely a result of PD progression, but may shape localised nigrostriatal neuronal degeneration in PD. Several studies utilising 1-methyl-4-phenyl-1,2,3,6-tetrahydropyridine (MPTP; an established PD toxin model) demonstrate that TLR4 knock-out in mice attenuates the MPTP-induced inflammation, PD pathology and symptoms [62–64]. Although α-synuclein accumulation is still evident in the midbrain after MPTP administration, there is no corresponding increase in dopaminergic neuronal death, indicating a putative role of TLR4 in mediating dopaminergic cell loss in response to environmental toxins and α-synuclein pathology [65]. Given the established roles of TLR2 and TLR4 in innate immunity, coupled with the inflammatory and neurodegenerative nature of PD, the preceding evidence concerning alterations of TLR2 and TLR4 in PD, especially in the CNS, is not surprising. However, viewing PD gut dysfunction in light of the increasing evidence concerning the diverse and important roles of TLR2 and TLR4 in normal gut functioning provides an interesting scaffold to understand the gut-driven PD pathogenesis, and may provide early preventative or improved therapeutic targets.

## TLR2 and TLR4 regulate interactions between the gut microbiome and intestinal barrier

Gastrointestinal TLRs have wide influence on gut physiology, regulating crypt dynamics, epithelial barrier integrity, mucosal immune responses (comprehensively reviewed in [3]), as well as the development and function of the ENS (which will be reviewed in the next section). TLRs 1–9 are expressed in a variety of gut cell populations, including epithelial cells, enteroendocrine cells and enteric neurons.

As previously mentioned, the gut lumen houses the gut microbiota, which has co-evolved with humans over millennia. The gut microbiota has been a topic of intense scientific effort and significance over the past two decades, and it is clear that the gut microbiota is integral to health, communicating with and influencing all aspects of human biology (e.g., microbiome-immune axis, gut-brain axis, gut-skin axis, etc.). Notably, studies utilizing germ-free (GF) animals born in sterile conditions without a gut microbiome or microbiota-depletion models using antibiotics have both been essential in establishing the influence of gut microbiota on development and overall health. For example, immune development, stress responsiveness, learning, memory as well as emotional and social behaviours are altered in GF rodent models [66–68]. Interestingly, Thy1-α-synuclein over-expressing mice, a familial model of PD, raised in GF conditions demonstrate reduced α-synuclein-dependent microglial activation with less motor and gastrointestinal deficits than mice colonised with a complex microbiota [69]. Similarly, oral antibiotic administration ameliorates dopaminergic neurodegeneration in 6-hydroxydopamine (6-OHDA; another established PD model) rodents, indicating that the microbial-driven signalling can exacerbate the central neurodegeneration of PD [70]. While these findings highlight the role of microbiota and microbial-driven inflammation in PD, as well as the complexity of the disease, further research is required to understand the contribution of the microbiome and population shifts in more clinically relevant studies.

The composition of the microbiota is constantly shaped by host genetics, environmental factors and lifestyle, and undergoes short-term oscillations (e.g. in response to timing or type of food and exercise [71]), mid-term variations (e.g. hormonal influence [72]) and more sustained shifts, most notably the age-associated dysbiosis [73]. Typically, the gut microbiome constitutes a diverse array of commensal microbes, and such microbial fluctuation is benign. However, certain endogenous or exogenous factors can encourage the growth of pathogenic microbes, leading to a dysbiotic microbiome which causes local gut inflammation with detrimental or harmful downstream consequences throughout the body [74–76]. Moreover, dysbiosis and gut inflammation can perpetuate each other, resulting in a vicious pro-inflammatory cycle [77, 78].

**Fig. 1:**
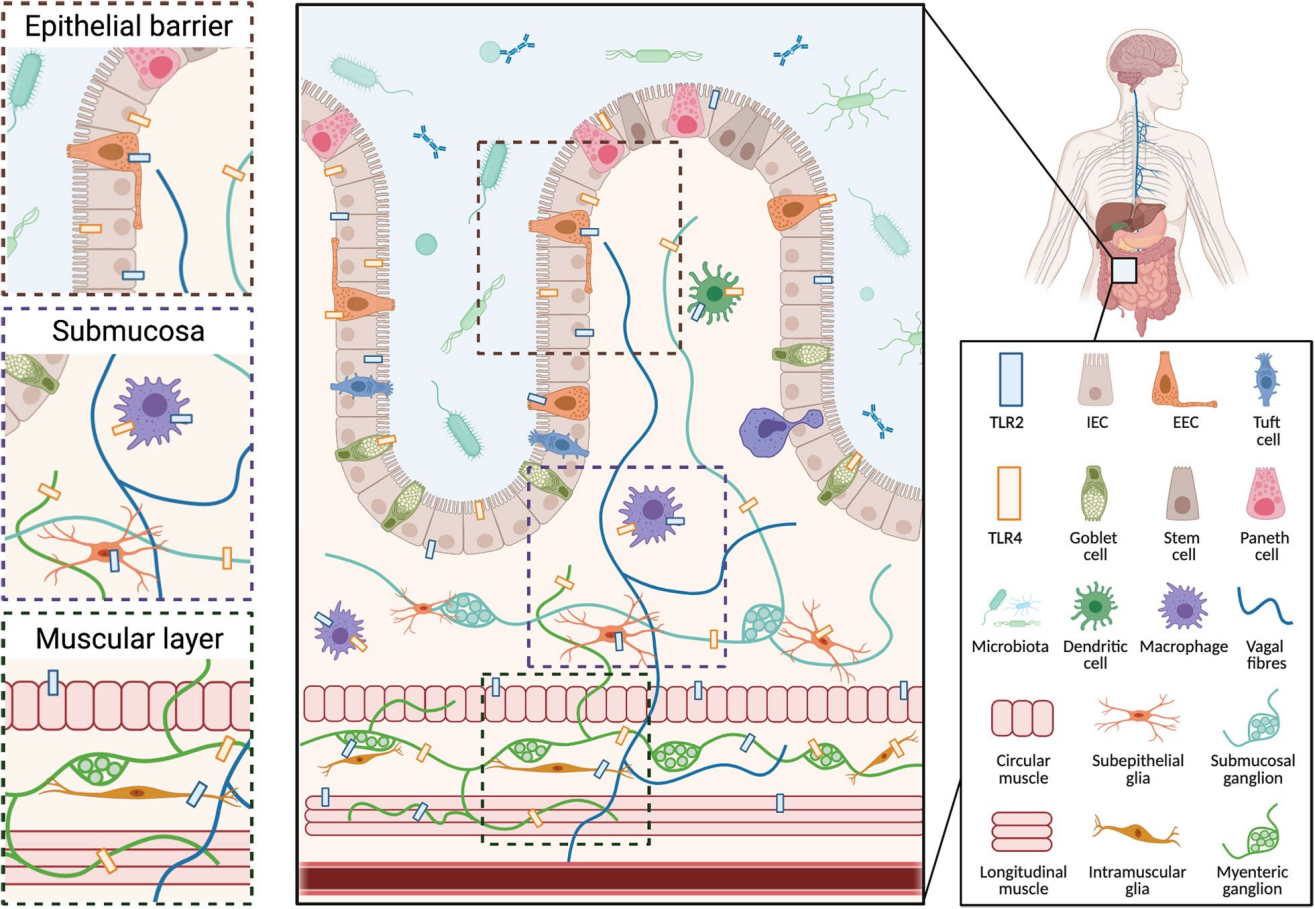
Representative schematic of TLR2 and TLR4 distribution in the gut. The Toll-like receptors 2 and 4 (TLR2 and TLR4) are expressed by many different cell populations throughout the gastrointestinal barrier, where they are activated by microbial metabolites and endogenous molecules. TLR2 and TLR4 are expressed by intestinal epithelial cells (IECs) and enteroendocrine cells (EECs) in the epithelial barrier, macrophages, dendritic cells in the submucosa, and smooth muscle cells in the muscular layer of the gut and throughout the enteric nervous system (subepithelial and myenteric, neurons and glia) [37, 81, 125, 126]. In addition to a role in innate immunity, TLR2 and TLR4 in the gut can regulate homeostasis and permeability, and influence motility through their effect on the enteric nervous system [3]. Figure created with Biorender.com

The intestinal epithelium is a selectively permeable barrier comprised of a single layer of epithelial cells interspersed by intraepithelial lymphocytes and enteroendocrine cells, which is lined by layers of mucous and antimicrobial peptides. Together, this gut barrier separates the intestinal milieu from the underlying lamina propria, and thus the rest of the body. Intestinal epithelial cells (IECs) include epithelial, goblet and Paneth cells, which are all critical for the development and maintenance of a healthy gut barrier [79]. The gut barrier is dynamically regulated by microbial metabolites and pattern recognition receptor signalling to simultaneously respond to symbiotic gut microbes and invading pathogens [80, 81]. Consequently, IECs express a variety of pattern recognition receptors, including TLRs, which form one of the first contact points between the external environment and internal biology. Here, TLR signalling controls crypt dynamics, intestinal permeability, antimicrobial molecule production as well as cytokine and chemokine secretion (Refer to Fig. 1 for representative diagram of TLR2 and TLR4 at the gut barrier). Consequently, altered TLR signalling (through receptor priming, protein levels and functioning) in IECs and the gut more broadly can be a cause for or a consequence of dysbiosis and leaky gut. These processes may occur in parallel independently, or in response to an alternative trigger [3].

It is widely accepted that microbial dysbiosis is evident in people with PD [9–13]; however, the dynamic nature of the microbiota, the complexity of microbe–host interactions and the heterogenous nature of PD complicates the understanding of which microbial changes directly contribute to the pathogenesis of PD, which are a result of ongoing disease processes or disease-associated lifestyle changes, or which may simply reflect an individual’s unique microbial “fingerprint”. Moreover, not everyone with a dysbiotic microbiota will develop PD. Such complexity only underscores the necessity to investigate aspects of host biology which may mediate a dysbiotic, pro-inflammatory cascade. Indeed, the role of TLRs as mediators of the microbiome–gut brain axis and innate immune system in PD has been elegantly reviewed previously [4], whereas this piece focuses on the role of TLR2 and TLR4 in gut physiology overall and a possible contribution to early PD pathogenesis through a local effect on gut and ENS function.

### TLR2 at the gut barrier

TLR2 expression in the gut barrier is shaped by the gut microbiome, with low levels in small intestine crypts and villi and higher expression in the proximal colon [81]. Notably, TLR2 signalling is crucial for the development and remodelling of the intestinal epithelium and mucosa. Oral administration of synthetic lipopeptide to mice influences intestinal goblet cell proliferation and size in a TLR2-dependent manner [82], and TLR2 knock-out impairs the proliferation cycle of epithelial cells after microbial colonisation in mice [83]. Throughout life, TLR2 activation by commensal microbes promotes gut homeostasis through increased microbial tolerance and epithelial barrier integrity. In healthy people, TLR2 signalling increases the translocation of tight junction proteins zonulin and occludin to epithelial tight junctions [84]. However, oral sugar tests and serum endotoxin levels in people with PD indicate elevated intestinal permeability [14], attributed to altered zonulin and occludin localisation in the intestinal epithelial barrier [15, 16]. Given that exposure of cultured IECs to LPS from enteropathogenic *Escherichia coli* (*E. coli*) increases both TLR2 and TLR4 expression [85] and that stimulation of IECs with peptidoglycans causes TLR2 trafficking from apical to basolateral membranes [84], it is possible that a dysbiotic gut microbiome alters intestinal TLR2 levels and thus contributes to early gut dysfunction in people with PD. Conversely, exposure of cultured human colorectal cells and mice with induced colitis to various commensal *Lactobacillus* strains exerts a beneficial influence on intestinal barrier function in a TLR2-dependent manner [86, 87]. Similarly, exposure of cultured intestinal epithelial cells to commensal *Bacteroides fragilis* decreases *TLR2* mRNA expression levels and increases anti-inflammatory cytokine production [85]. A recent study demonstrated that TLR1/TLR2 and TLR2/TLR6 signalling cascades are inhibited by TLR10, and probiotic treatment to IECs in vitro altered TLR2-TLR10 signalling pathways to activate the anti-apoptotic phosphatidylinositol 3-kinase (PI3K)/protein kinase B (AKT) signalling, inhibit NF-κB signalling and improve intestinal barrier permeability by altering zonulin localisation [88]. Further studies are required to determine if similar TLR2-dependent changes to tight junction proteins and microbial tolerance occur in people with PD, thus opening the door to targeted probiotic treatments.

#### TLR2 detection of bacterial amyloids mediates α-synuclein aggregation in PD

Many bacteria produce functional amyloids, which are insoluble extracellular proteins that support bacterial growth and survival. However, amyloids activate the innate immune system through TLR1 and TLR2, and are also capable of cross-seeding the misfolding of endogenous host proteins into amyloid fibrils [89]. For example, activation of TLR2/TLR1 heterodimers by curli fibrils in intestinal epithelial cells in vitro increases the permeability of the intestine and bacterial translocation [90]. Consequently, bacterial amyloids have been implicated in PD pathogenesis. Specifically, the amyloid curli protein produced by *E. coli* accelerates α-synuclein fibrilization in amyloid assays in vitro, and exacerbates α-synuclein-induced pathology in mice overexpressing human α-synuclein (Thy1-αSyn Line 61), including motor impairments, altered bowel motility, as well as phosphorylated α-synuclein deposition and microglial activation in the midbrain [91]. These results are not evident in wild-type mice, demonstrating that exposure to amyloid curli proteins alone is not sufficient for PD pathology but requires another contributing factor such as elevation of α-synuclein [91]. Furthermore, aged rats treated with oral amyloid-producing *E. coli* demonstrated increased α-synuclein deposition in submucosal and myenteric ganglia, coupled with central microgliosis, astrogliosis, increased TLR2 expression and α-synuclein aggregation throughout the brain, despite no evidence of bacterial amyloid infiltration into the brain or other tissues [92]. Although this study did not investigate intestinal TLR2 expression or intestinal inflammation, it supports a role of gut-derived bacterial products and TLR2 in immune priming and α-synuclein pathology in PD. Moreover, it highlights the potential for bacterial curli to cross the intestinal barrier, increase the expression of endogenous protein and cross-seed protein misfolding in the host [92], leading to enteric α-synuclein pathology. Further studies are required to investigate the influence of the bacterial amyloid curli protein on intestinal or enteric TLR2 signalling and in animal models of sporadic PD, especially in the context of a leaky gut when curli proteins could readily cross the intestinal barrier to interact with endogenous α-synuclein from enteric neurons. Moreover, as many bacteria release both LPS and curli proteins, it would be interesting to investigate a cumulative effect of LPS-TLR4 and curli-TLR2 signalling in gut inflammation and PD pathology.

### TLR4 at the intestinal barrier

TLR4 expression in the gut is dynamic during development and throughout life, influenced by immune and microbial signals as well as lifestyle factors [93–95]. Reflecting the increased bacterial load along the digestive tract, TLR4 expression is typically low in enterocytes and Paneth cells of the small intestine but high in colonocytes, enteroendocrine cells and goblet cells of the colon [3, 81]. TLR4 has a complex regulatory influence on many aspects of the gut epithelium, notably the permeability of the intestine and the integrity of the mucosa. For example, TLR4 activation impairs the proliferation of IECs in vitro and of ileal enterocytes in newborn, but not adult, mice [96]. Similarly, administering LPS to human-derived Caco-2 cell monolayers (colon epithelial cells) in vitro as well as to mice has been shown to increase intestinal permeability, which is mediated by increased enterocytic expression of TLR4 and its increased co-localisation with co-receptor CD14 [97]. Such increase in intestinal permeability may arise due to an influence on tight junction proteins, as LPS treatment of cultured IECs alter the localisation of tight junction protein zona occludens 1 in a TLR4-dependent manner, leading to increased paracellular permeability and detachment of adjacent cells [98]. Overexpression of epithelial TLR4 in transgenic mice reduces the expression of epithelial cell adhesion and tight junction protein genes, leading to an altered gut microbiome and increased bacterial translocation into circulation [99]. The microbe–TLR4 signalling also shapes the differentiation of epithelial cells and thus intestinal mucous production. For example, the maturation of murine proximal colonic crypt organoids is LPS-dependent, where the LPS–TLR4  signalling impairs the proliferation of the intestinal epithelium but enhances the differentiation of certain lineages, especially goblet cells [100]. Evidently, the expression and activation of intestinal TLR4 is a carefully orchestrated process, and alterations to this balance can have widespread effects on gut function and homeostasis.

#### TLR4 at the gut barrier mediates PD risk factors

TLR4 signalling at the gut barrier may mediate the contributions of genetic, lifestyle and environmental factors to the risk of PD. First, a genetic variant of *TLR4* (rs1927914) is associated with the risk of sporadic PD [101], and further stratifies PD risk when comparing individuals who drink alcohol to those who do not [44]. Diets high in fat, processed food and red meat (e.g. typical Western diet) are linked with increased PD risk [102, 103], and TLR4 is central to the inflammation and endoplasmic reticulum stress resulting from high-fat or high-cholesterol diets [104, 105]. Interestingly, high-fat diets specifically increase the expression of intestinal TLR4 [95], and exacerbate intestinal barrier dysfunction in a TLR4-dependent manner in rats with severe acute pancreatitis [106]. Such diets not only encourage the growth of gram-negative, LPS-producing bacteria [107], but also increase the amount of lipids and free fatty acids, dietary components which, by themselves, can modulate TLR4 signalling [108] or rely on TLR4-priming for consequent lipid-induced inflammation [109].

TLR4 expression is also altered in a variety of PD models. For example, striatal expression of TLR4 is increased in rodents after intra-striatal infusion of neurotoxin 6-OHDA, bacterial LPS and viral polyinosinic:polycytidylic acid (Poly I:C) [110]; as well as intraperitoneal and intranasal LPS administration [111]. While these methods of infusion are not directly clinically relevant, these afore-mentioned PD factors would usually enter the body via the gut, where they influence the gut microbiome and intestinal epithelium before having systemic effects. Accordingly, chronic, low-dose administration of LPS via drinking water exacerbates PD pathology in α-synuclein-overexpressing mice, supporting a role of TLR4-mediated gut inflammation in disease progression [112]. Moreover, chronic stress (through an unpredictable restraint model) in a low-dose oral rotenone PD mouse model exacerbates CNS pathology and gut dysfunction compared to rotenone alone [113]. This study reports elevated striatal TLR4 expression, demonstrating that exposure to toxins in the gut could interact with the psychological/physiological state and elevate TLR4 expression in the brains of people with PD. Similarly, TLR4-knockout in mice reduces intestinal and neuro-inflammation, neurodegeneration and PD-like symptoms after oral rotenone administration [60]. Taken together, these studies indicate that genetic, environmental and lifestyle factors can contribute to PD pathology via TLR4 signalling in the gut and along the gut–brain axis. However, the underlying mechanism is still unclear, as research in clinically relevant models with luminal LPS exposure and intestinal TLR4 activation is still lacking.

#### Intestinal epithelial cells, TLR4 signalling & inflammation in PD

Excessive or altered TLR4 signalling may contribute to the gut inflammation present in PD. Importantly, people with PD exhibit high serum levels of LPS-binding protein coupled with increased intestinal permeability and epithelial staining of α-synuclein [14]. This is accompanied by an overabundance of gram-negative, LPS-producing bacteria in the gut microbiome of people with PD [112, 114, 115], and elevated epithelial TLR4 levels in PD colonic biopsies coupled with increased cytokine expression and dysbiosis [60], indicating a link between microbial LPS, a leaky gut, TLR4 signalling and intestinal PD pathology. However, it is still unknown if the increased expression of TLR4, or altered TLR4 signalling, drives early disease processes or arises from PD-associated dysbiosis and inflammation.

In the gut, TLR4 levels are regulated by many different mechanisms in response to acute or chronic stimulation, or long-term epigenetic signalling. IECs usually express low levels of TLR4, which are concentrated on the basolateral side to avoid excessive immune activation at the gut barrier in response to luminal gram-negative bacteria and LPS [116]. Chronic LPS exposure causes cellular internalisation of TLRs (including TLR4) to prevent excessive pro-inflammatory signalling [117]. Furthermore, in healthy IECs, in vitro administration of LPS or commensal bacteria increases the expression of peroxisome proliferator-activated receptor-γ (PPARγ), which inhibits the TLR-mediated intestinal inflammation [118]. This homeostatic mechanism appears to be absent in people with IBD, as increased TLR4 expression coupled with decreased expression of PPARγ and Tollip (another TLR-inhibitory protein) were evident in intestinal biopsies of people with active ulcerative colitis [118]. Alternatively, low TLR4 levels can also be driven by DNA methylation of the *TLR4* promoter region [94, 119], and DNA methylation patterns are strongly influenced by diet and the gut microbiome [120–123], suggesting the gut environment shapes TLR4 signalling both in the short term and over one’s lifetime.

TLR4 levels throughout the intestinal epithelium and in the wider body are also elevated in PD [48, 60], indicating perturbed regulation. Given the similarities between IBD and PD, the influence of diet and gut microbiome on DNA methylation [120–123], and that many PD risk factors can cause intestinal dysbiosis, it is conceivable that dysbiosis simultaneously increases LPS levels (through the growth of gram-negative bacteria) and responsiveness (e.g., altering TLR4 or inhibitory PPARγ levels), leading to excessive LPS-TLR4 signalling and inflammation in the gut. Further research is required to establish a link between the gut microbiome, PD factors and TLR4 signalling in the gut with PD pathology. Although pre-existing polymorphisms in *TLR4* have been linked to PD, it is unclear if other genes that influence the signalling of TLR4 (e.g., PPARγ and Tollip) or regulate exposure to various pro-inflammatory microbial metabolites (e.g., tight-junction proteins which mediate intestinal permeability) also influence PD risk or progression. Additionally, while the interaction between TLR4 and LPS is most studied, it is important to remember that TLR4 interacts with a variety of other PAMPs and DAMPs (including α-synuclein), and further studies are required to characterise other possible mechanisms linking TLR4 activation to dysbiosis and intestinal inflammation in PD.

## TLRs and the ENS

The ENS is a complex, interconnected network of neuronal and glial circuits located along the digestive tract, which exerts intrinsic control on gut motility and secretion, and is a critical mediator of host–microbe interactions. The ability to function independently from CNS control has earned the ENS the title of ‘second brain’. However, vagal, pelvic, sympathetic and parasympathetic inputs feed into the myenteric and submucosal plexuses, and the ENS sends afferent projections to the brain. Together, this forms the physical network of the bidirectional gut-brain axis, which is also closely tied to the immune system [124]. The gut microbiome is crucial for post-natal ENS maturation, and mounting evidence demonstrates that the plasma-membrane TLR2 and TLR4 on enteric neurons and glia shape neurogenesis of the ENS, as well as enteric neuronal survival and signalling [125, 126]. Moreover, aberrant ENS development and function are linked with a number of bowel conditions such as enteric neuropathies, IBS, IBD and colorectal cancer (recently reviewed in [124]), and the gut dysfunction and dysbiosis evident in PD suggest a role for the ENS in PD pathology. However, ENS research is still a growing field, especially in terms of dysfunction and disease.

### TLR2 influences the development and function of the ENS

Microbial-TLR2 signalling influences neurogenesis throughout the body. In the brain, administration of bacterial lipopeptides (TLR2 agonists) to cultured neuronal progenitor cells inhibits neurosphere formation and the proliferation of neural progenitor cells in the telencephalon of mice, indicating that TLR2-mediated inflammation arising from bacterial signals influences brain development [127]. Furthermore, TLR2 is expressed by enteric neural progenitor cells, enteric neurons and enteric glial cells [128, 129]. Microbial signals can regulate the expression of intestinal [93] and striatal TLR2 [130], and TLR2 activation also influences the ENS. Due to its proximity to the single-cell intestinal epithelium, the ENS is continuously exposed to the gut microbiome and other luminal contents including dietary components and antibiotics. Consequently, the ENS is in a continuous cycle of neuronal loss and replacement throughout life [131], with a study demonstrating that TLR2 promotes colonic neurogenesis in adult mice in a gut microbiome-dependent manner [128]. Furthermore, exposure to pathogenic *E. coli* and probiotic *Lactobacillus paracasei* F19 bacteria increases TLR2 expression in enteric glial cell cultures [132], whereas administration of dextran sulfate sodium to TLR2 knock-out mice alters enteric neurochemical profile and architecture, reduces intestinal motility and mucosal secretion, and causes the TLR2 knock-out mice to develop more severe colitis than wild-type mice [125], highlighting the importance of the signalling between microbes and TLR2 for ENS function.

### TLR4 promotes ENS survival

TLR4 is expressed by both enteric neural progenitor cells [128] and enteric neurons [129], where it plays an important role in neuronal survival and motility. In mice, loss of TLR4 signalling through genetic models, or as a consequence of microbiome depletion results in delayed gastrointestinal motility and reduces nitrergic neuronal counts, with subsequent in vitro experiments demonstrating that LPS exposure improves primary enteric neuron survival in a TLR4-dependent mechanism [133]. More recently, TLR4 activation by LPS administration during antibiotic treatment prevents the antibiotic-induced enteric neuronal loss in mice, but has no benefit if administered 14 days after antibiotic treatment, supporting a role for LPS-TLR4 signalling in enteric neuronal survival, but not in neurogenesis or differentiation [134]. TLR4 is also expressed by enteric glial cells [132], which protect enteric neurons and facilitate ENS signalling, and TLR4 knock-out mice exhibit altered ileal dysmotility and reactive gliosis [135, 136]. Moreover, TLR4 signalling may mediate the negative influence of poor diet on ENS health. Notably, mice given a ‘Western diet’ (35% kcal from fat, enriched in palmitate) for six weeks demonstrated increased faecal LPS levels and plasma free fatty acids, nitrergic myenteric cell loss and colonic dysmotility, which were not evident in TLR4 knock-out mice [137]. Evidently, TLR4 signalling is crucial for normal ENS function, however, understanding of how impaired or excessive TLR4 signalling influences the ENS is still in its infancy.

### ENS in PD

Although some people with PD exhibit enteric α-synuclein aggregates and Lewy neurites [138, 139] as well as enteric neuronal inflammation [140], conflicting reports exist concerning enteric denervation [139, 141, 142]. Exposure to PD-associated factors also alters the ENS, as intraperitoneal MPTP injection selectively reduces the dopaminergic neuronal density in myenteric ganglia, but does not affect the cholinergic or nitric oxide neurons [143]. As enteric dopaminergic neurons regulate the contractile activity of the rodent gut via D_1_-like receptor signalling which generates colonic peristalsis, it is hypothesised that changes to gut motility and thus constipation in people with PD arise from the denervation of enteric dopaminergic neurons [144]. However, control of gut motility is a complex process, and other research implicates an impairment of cholinergic signalling driving gut dysmotility after exposure to PD-related factors. For example, transgenic mice overexpressing human α-synuclein with a PD-associated mutation (A53T) display α-synuclein aggregates in the myenteric and submucosal plexi with reduced motor responses in longitudinal and circular muscle layers, arising from impaired cholinergic transmission in the ENS [145]. Similarly, dysbiosis induced by Western diet consumption causes  colonic nitrergic myenteric cell loss [137], further highlighting the importance of PD-associated environmental factors and the microbiome in ENS health. In light of gut dysbiosis in people with PD and the interrelationship between the ENS and gut microbiome, further studies are required to clarify the extent of ENS denervation in PD, and if this is a cause or a consequence of gut dysfunction in PD.

Such research is complicated by inherent clinical heterogeneity of PD coupled with the bidirectional relationship between intrinsic (ENS) and extrinsic (CNS) control of gut function. For instance, nigral overexpression of α-synuclein or nigrostriatal dopaminergic neurodegeneration can induce ENS changes via the gut-brain axis [146, 147], such as submucosal neuronal loss and increased myenteric glial expression [148]. Similarly, unilateral injection of 6-OHDA into the medial forebrain bundle of mice can result in downstream colonic dysmotility and enteric denervation in the distal colon [149]. Studies demonstrating the complex nature of CNS-ENS signalling support the notion of PD subtypes, where central denervation may drive gut dysfunction in some, and enteric denervation in others. As such, future studies grouping people with PD by clinical presentation and other features may help clarify the causes of enteric degeneration and gut dysfunction (including dysphagia, slowed gastric emptying and constipation) in different PD subtypes. Accordingly, IBD is associated with ENS abnormalities [150], and it is likely that hypotheses regarding gut inflammation contributing to α-synuclein pathology in the ENS apply  to a subset of people with PD. Enteric neurons are the main cell population expressing α-synuclein in the gut, and given the proximity of the ENS to the gut lumen coupled with the critical role of TLR2 and TLR4 signalling in enteric innervation and function discussed above, further studies are required to investigate the contribution of TLR2 and TLR4 to  ENS changes and α-synuclein pathology in the context of PD. Moreover, investigation of TLR2, TLR4 and α-synuclein in the enteric glia is also critical.

## TLRs and α-synuclein

### α-synuclein as an immune signalling molecule in PD

A number of neurodegenerative diseases are attributed to protein misfolding and consequent aggregation in the nervous system, leading to neurotoxicity and cell death. Protein misfolding occurs due to a complex interaction between environmental, genetic and age-related factors which influence the physicochemical state of the body, leading to impaired protein synthesis, folding and clearance [151]. In PD, the main component of pathogenic Lewy bodies and Lewy neurites is α-synuclein, a natively unfolded protein with conformational flexibility, existing as monomers and oligomers in healthy cells where it is involved in neurotransmitter release, synaptic transmission, and mitochondrial and lysosomal function [152]. To date, α-synuclein accumulation is considered an abnormal or dysregulated process, arising from a combination of altered gene expression and impaired protein degradation. These insoluble α-synuclein aggregates can be released from neurons in a variety of ways, including exosome-mediated secretion or during apoptosis. In the extracellular space, α-synuclein can seed the misfolding of surrounding proteins or is rapidly taken up by neighbouring cells [153, 154] and transported retrogradely along neuronal axons [155, 156]. Interestingly, emerging evidence suggests that α-synuclein has immune-related functions where the afore-mentioned folding, release and uptake may be useful molecular mechanisms rather than pathogenic processes in themselves. As such, viewing elevated α-synuclein levels and consequent PD pathology through the lens of the protein’s immune signalling provides an interesting framework to link together various elements occurring in some people with idiopathic PD, including risk factors, gut dysfunction and microbial dysbiosis.

**Fig. 2:**
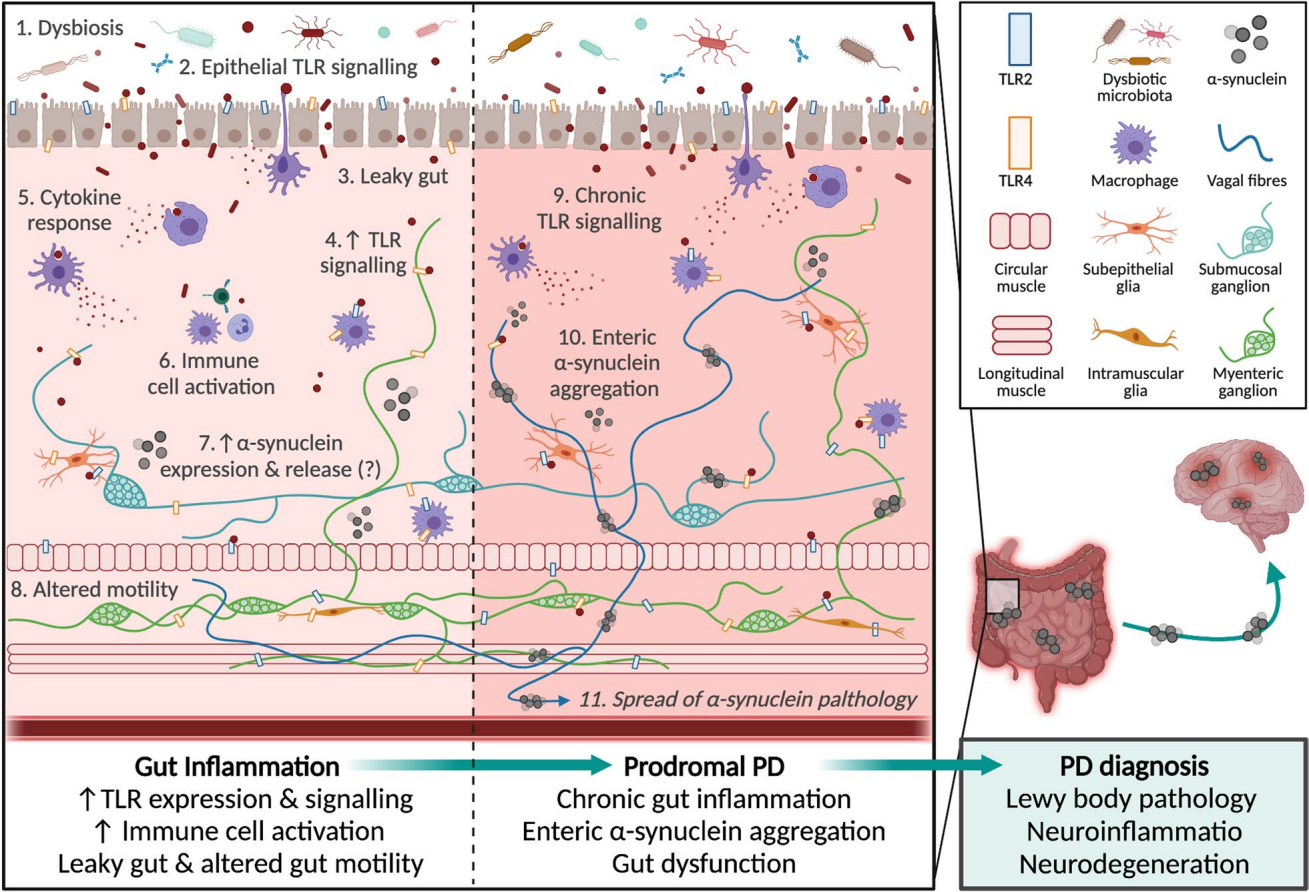
Representative schematic of TLR-driven gut dysfunction which may contribute to PD. Microbial dysbiosis and pro-inflammatory gut responses can result in a complex positive feedback loop of gut leakiness and inflammation. For example, microbial dysbiosis (1) contributes to the signalling of epithelial toll-like receptors (TLRs) (2), which can result in a leaky gut (3) and further TLR signalling (4) with subsequent cytokine secretion (5) and immune cell activation (6), leading to TLR-associated dysbiosis [3, 4]. Such gut inflammation can alter the enteric nervous system signalling and result in altered motility (7) [135]. It is hypothesised that α-synuclein is secreted by enteric neurons in response to infection to function as an immune-signalling molecule [159], and α-synuclein can directly activate and also change the expression of TLR2 and TLR4, causing further inflammatory responses [57, 58]. Over time, chronic TLR signalling, gut inflammation and sustained α-synuclein secretion may contribute to the formation of insoluble α-synuclein aggregates in the gut, especially if combined with other molecular deficits (e.g., lysosomal dysfunction). This may cause gut dysfunction (e.g. constipation), which is increasingly recognised as a ‘prodromal PD’ phase [20, 36]. Studies demonstrate that insoluble α-synuclein aggregates can spread from the gut to the brain via peripheral nerves (e.g., vagus nerve), where they cause Lewy body pathology in the CNS, neuroinflammation and neurodegeneration, resulting in the characteristic motor impairments of PD [27–30]. Figure created with Biorender.com

Although largely studied for a role in neuronal synaptic communication, recent studies propose α-synuclein functions as an immune signalling molecule [157]. Extracellular α-synuclein exerts a chemoattractant influence on human neutrophils and monocytes and stimulates dendritic cell maturation in vitro [158], and α-synuclein functions as a natural antimicrobial peptide [159, 160] and inhibitor of viral replication [161]. Notably, α-synuclein is an endogenous agonist for TLR2 and TLR4 [51, 57], which will be discussed in sections below. Consequently, it is hypothesised that α-synuclein is mobilised in response to pathogens by central and peripheral neurons, and enteroendocrine cells in the gut epithelium [152, 162, 163]. Accordingly, the mucosa and enteric plexuses of the human appendix store an abundance of aggregated and oligomeric α-synuclein [164], and the level of α-synuclein staining within enteric neurons correlates with the degree of inflammation in human intestinal biopsies [158]. Despite the uncertainty regarding the trafficking, release and immune-related role of α-synuclein, it is evident that α-synuclein expression increases in response to pro-inflammatory triggers, and changes of the surrounding extracellular micro-environment (e.g., temperature, pH, metal ions, metabolites) can impact the conformation of α-synuclein to cause protein folding. In turn, the misfolded α-synuclein forms pathological oligomers, fibrils and Lewy bodies, which cause lysosomal and mitochondrial dysfunction, interfere with microtubule formation, vesicular homeostasis and autophagy, and activate microglia [165, 166]. Consequently, not only could a leaky gut and resulting exposure to translocating pathogens trigger increased α-synuclein levels within certain gut cells, but prolonged inflammation and gut pathology may also encourage misfolding and aggregation of α-synuclein (Fig. 2), especially when coupled with genetic overexpression of α-synuclein or disrupted protein clearance [167]. Furthermore, the high apoptotic turnover of certain gut cell populations means that insoluble α-synuclein aggregates could be regularly and chronically released from enteroendocrine cells into the extracellular space of the gut barrier. As previously mentioned, α-synuclein can be secreted and taken up by various cell types in a number of ways, meaning that misfolded α-synuclein in the gut barrier can readily spread into adjacent enteric neuronal fibres, to seed the misfolding of surrounding proteins and trigger further inflammation and neurodegeneration. Consequently, gut inflammation could perpetuate a cycle of elevated α-synuclein levels, misfolding and inflammation in the local environment, which may contribute to PD. This hypothesis is supported by reports that prior gastrointestinal infection is associated with future PD risk [168], and Lewy body pathology is evident in the gastrointestinal tract of people years prior to their PD diagnosis [169]. Interestingly, *Helicobacter pylori* infections after PD diagnosis can worsen the motor symptoms [170], suggesting a role for infection-mediated gut dysfunction in both PD pathogenesis and progression.

In addition, prior viral infection outside the gastrointestinal system may also be associated with the increased risk of developing PD, supporting hypotheses that the immune-related role of α-synuclein is an endogenous, physiological cause of increased protein levels, especially in people with sporadic PD. For example, α-synuclein levels are increased in the substantia nigra of some individuals with human immunodeficiency virus (HIV) [171] and infection with Western equine encephalitis virus causes α-synuclein aggregation with selective dopaminergic loss in mouse brains [172]. Such elevated levels of α-synuclein are thought to be a direct immune response to the viral pathogen, and may explain the link between prior infections and PD risk in humans. For example, infection with West Nile virus increases α-synuclein protein levels in primary striatal neurons in vitro, while α-synuclein-knockout mice exhibit higher viral load and mortality after infection with West Nile virus and Venezuelan equine encephalitis virus in comparison to wild-type and heterozygous littermates, supporting a role of α-synuclein in the inhibition of viral replication and associated viral pathology [161]. Of particular note, growing numbers of acute parkinsonism following Severe Acute Respiratory Syndrome Coronavirus 2 infection are being reported [173, 174], and coronavirus disease 2019 (COVID-19) affects both the olfactory (anosmia) and the gastrointestinal tracts (microbial dysbiosis, diarrhoea and colonic inflammation) in some people, reflecting Braak’s two hypothesised PD initiation sites [175]. Furthermore, a number of molecular similarities have been identified between COVID-19 and PD, including oxidative stress, inflammation and protein aggregation [175]. Although there is currently insufficient longitudinal data to link COVID-19 infection or “long COVID” with a risk of developing PD, future research on the associations between viral infections, TLR signalling, α-synuclein and PD is critical in light of the ongoing pandemic.

In regards to PD, α-synuclein aggregates have been reported in gastrointestinal tissues, the ENS, and the vagus nerve of people with PD [176, 177], even before motor impairments or clinical diagnosis of the disease [169, 178]. However, gastrointestinal and enteric α-synuclein aggregates are not exclusive to people with PD [179, 180]. Although this non-specificity diminishes the potential of enteric α-synuclein aggregates for early PD diagnosis, it provides important information regarding early disease processes in the gut. For instance, elevated levels of α-synuclein are evident in the colonic myenteric plexus of people with Crohn’s disease [181] and in enteric neurites from endoscopic biopsies of children with intestinal inflammation, where the levels of α-synuclein correlate with the degree of intestinal inflammation as measured through CD68 staining [158], thus strengthening the link between dysbiosis, intestinal inflammation and α-synuclein pathology. Interestingly, although elevated levels of α-synuclein are evident in 52% of post-mortem colonic ENS samples from healthy people without a neurodegenerative diagnosis, they are not present in any samples from those with Alzheimer’s disease (*n* = 8) [138], highlighting the role of gut inflammation and α-synuclein in PD specifically rather than in all forms of neurodegeneration. In light of the growing evidence linking gut inflammation and α-synuclein, it has been proposed that the previously reported propagation of α-synuclein from the gut to the brain via the vagus nerve [27, 29] may represent an immune signal to prepare the CNS against future infections rather than a pathogenic prion-like transmission of the disease [159]. Accordingly, a single intraperitoneal injection of LPS to mice increases levels of α-synuclein and phosphorylated α-synuclein in colonic myenteric neurons without associated α-synuclein pathology in the brain, or corresponding motor symptoms [182]. Consequently, early gut dysfunction arising from disrupted TLR2 and TLR4 signalling could directly contribute to the elevated levels and aggregation of α-synuclein in prodromal PD. Moreover, as α-synuclein is an endogenous agonist of both TLR2 and TLR4, it is conceivable that elevated TLR levels or TLR priming due to pre-existing inflammation can feed into a positive feedback loop of inflammation-induced α-synuclein–TLR signalling.

### α-Synuclein and TLR2

Studies have also demonstrated a histopathological and functional molecular link between TLR2 and α-synuclein. Notably, TLR2 co-localises with α-synuclein in Lewy bodies from the anterior cingulate cortex of post-mortem brain tissues from people with PD [50]. On the cellular level, α-synuclein is an endogenous TLR2 agonist, and directly causes microglial activation with upregulation of TLR2 expression [58]. TLR2 activation increases neuronal, astrocytic and microglial uptake of α-synuclein fibrils [50], with different underlying mechanisms and consequences [183]. In neurons and astrocytes, TLR2 stimulation impairs α-synuclein fibril degradation [183], and treatment of SH-SY5Y and primary human neuronal cultures with the TLR2 agonist PAM3CSK4 (but not the TLR4 agonist, LPS) increases  α-synuclein levels, due to impaired lysosomal degradation or autophagy [50]. Correspondingly, TLR2 activation inhibits autophagic activation through AKT/mTOR activation, whereas inhibition of TLR2 signalling reduces neuronal α-synuclein aggregation in transgenic TLR2 knock-out mice overexpressing human α-synuclein [184]. Thus, neuronal and astrocytic TLR2 activation increases α-synuclein uptake and impairs degradation processes, contributing to neurotoxicity. Conversely, cell-released α-synuclein activates microglia, where α-synuclein–TLR2 stimulation is required for pro-inflammatory responses and α-synuclein internalization [51] but the degradation occurs in a TLR2-independent manner [183]. Exposure of cultured microglia to recombinant α-synuclein activates the cells and increases TLR2 expression [58]. In addition to cell-type differences in the α-synuclein–TLR2 responses, the conformation of α-synuclein also plays a role. Investigation of primary rat microglial cultures and TLR2 knock-out mice indicates that oligomeric α-synuclein secreted from neurons causes microglial activation in a TLR2-dependent manner [51], while the fibrillar α-synuclein aggregates (but not oligomeric) cause inflammation via TLR2 signalling in an immortalised human monocyte cell line [185], indicating cell- and conformation-specific responses to α-synuclein. Similarly, a recent paper which exposed microglia to different α-synuclein conformations in vitro demonstrated that α-synuclein monomers and oligomers activate the NLRP3 inflammasome via TLR2 and TLR5, whereas fibrils cause NLRP3 activation after initial priming with LPS and the “ribbon” conformation does  not activate microglial inflammasomes, where the NLRP3 inflammasome is important for α-synuclein phagocytosis and breakdown [186]. Targeting TLR2 may be an effective treatment option for PD, as blocking TLR2 using a functional inhibitory antibody reduces the accumulation and the between-cell transmission of α-synuclein in neuronal and astroglial cell cultures, and ameliorates neuroinflammation, neurodegeneration and behaviour impairments in α-synuclein-overexpressing mice [187]. Further studies are required to investigate the role of TLR2 in the gut-brain propagation of α-synuclein, and the interaction between TLR2 and α-synuclein in immune cells, enteric neurons and gut cells such as enteroendocrine cells.

### α-Synuclein and TLR4

Extracellular α-synuclein is known to activate TLR4 in astrocytes and microglia [57, 188], and prolonged exposure of cultured human astrocytic and microglial cells to low levels of α-synuclein oligomers increases the responsiveness of TLR4 [52], thus causing a predisposition to an enhanced inflammatory response on exposure to TLR4 agonists such as microbial LPS. Further studies are required to investigate the effect of multiple inflammatory triggers such as LPS on α-synuclein levels, to determine if chronic dysbiosis or intestinal inflammation increases TLR4 or α-synuclein levels, leading to an augmented immune response with subsequent exposures. For instance, age-associated shifts in the microbial profile and immune system (“inflamm-aging”) are linked to age-related diseases [189, 190], and a recent study demonstrated that intranasal LPS infusion causes an increased inflammatory response and greater PD pathology in aged mice compared to young mice [191], although the expression or priming of TLR4 was not studied. Conversely, TLR4 is also required for microglial phagocytosis [57], and thus may contribute to the clearance of pathogenic α-synuclein [192]. Thus, it follows that TLR4 knockout in mice over-expressing oligodendroglial α-synuclein (a model of multiple system atrophy, another synucleinopathy) increases brain α-synuclein deposition, nigrostriatal dopaminergic neuronal loss and motor dysfunction, highlighting a potential neuroprotective role of TLR4 in α-synuclein clearance [193].

Taken together, it is evident that a complex relationship exists between α-synuclein and TLR4. Given that IBD, infections, antibiotics and diet may all contribute to intestinal inflammation and are also risk factors for PD, investigating the interactions between TLR4 and α-synuclein at the intestinal barrier may provide an important insight into early pathological processes that contribute to chronic inflammation and influence PD risk. Thus, TLR4 is an intriguing candidate mediating the link between intestinal inflammation and PD pathogenesis, and crucially the link with α-synuclein aggregation.

## Putative contribution of disrupted TLR signalling in PD pathogenesis

Increasing evidence indicates that α-synuclein interacts with both TLR2 and TLR4 to mediate immune activation and α-synuclein aggregation and clearance. Thus, TLR2 and TLR4 may be critical regulators of central or peripheral inflammation and α-synuclein pathology in PD, especially when considering that elevated α-synuclein alone is not sufficient to cause PD. For instance, pre-existing gut inflammation or increased α-synuclein expression and extracellular release can cause TLR activation with downstream immune responses, while prolonged inflammation can trigger α-synuclein misfolding into oligomers and fibrils, which also interact with TLR2/4. When coupled with other PD-associated biological disturbances (e.g., ageing, altered proteasome function, mitochondrial dysfunction), this may lead to α-synuclein pathology in the CNS or the ENS.

In the gut, TLRs, especially TLR2 and TLR4, are integral to normal gut homeostasis, promoting microbial tolerance and gut barrier integrity, and regulating immune responses to pathogens (Fig. 1). Various environmental, genetic and microbial factors can influence TLR signalling, and chronic exposure to pro-inflammatory factors (e.g., diet, infectious pathogens, antibiotics) can result in chronic inflammation, microbial dysbiosis and a leaky gut. Such gut dysfunction, especially the translocation of pathogenic microbes across the leaky gut barrier, is hypothesized to trigger α-synuclein release from certain gut cell populations as part of a normal immune response. In accordance with Braak’s hypothesis, α-synuclein aggregates have been reported to propagate from the gut to the brain via the vagus nerve. Here, α-synuclein aggregates trigger TLR2- and TLR4-mediated inflammation and microglial activation, eventually leading to neurodegeneration and the hallmark motor impairments of PD. Thus, it is conceivable that chronic, TLR-mediated gut dysfunction and inflammation may result in excessive α-synuclein expression throughout the gut and eventual pathological propagation to the brain (Fig. 2). Alternatively, local aggregation of α-synuclein in the gut arises from and contributes to a cycle of chronic gut inflammation and dysfunction, which triggers systemic inflammation. In turn, systemic inflammation can prime central immune receptors (including TLR2/4), alter the permeability of the blood–brain barrier and result in central neuroinflammation, which are directly thought to trigger central α-synuclein aggregation and neurodegeneration of the substantia nigra. Further research into the role of α-synuclein in the gut is required to verify these hypotheses and understand if and how a normal immune mechanism becomes pathogenic, leading to PD. Finally, it is important to acknowledge that some forms of PD may result from solely central neurodegenerative processes, and these seem to be associated with monogenic forms of PD with distinct pathology and symptoms compared to sporadic PD.

## Conclusion

PD is a complicated, multi-faceted disease with immense clinical and biological heterogeneity. Research concerning gut dysfunction in PD progression is continuing to grow, and there is increasing evidence that gut inflammation may contribute to PD pathogenesis in at least a subset of people with the disease, although the specific mechanisms are still unclear. Herein, we discussed the role of TLR2 and TLR4 in normal gut function, and presented evidence that disrupted TLR2 and TLR4 signalling, arising from inherent biological or external factors, may contribute to early gut dysfunction and evolution of the neurodegenerative processes in PD. Very few established genetic and environmental risk factors for PD are 100% predictive of the risk, whereas TLR2 and TLR4 expression, function and signalling are dynamic processes shaped by a multitude of genetic and environmental factors, many of which are implicated in PD. As such, TLR2 and TLR4 are likely candidates influencing gut leakiness and inflammation, enteric denervation and early colonic dysmotility, all of which may contribute to PD pathogenesis. Therefore, TLR2 and TLR4 may hold diagnostic potential to predict those at risk of PD or to stratify disease progression. Currently, most PD therapeutics are targeting the brain to improve symptoms of dopamine loss, and thus do little to prevent further neurodegeneration or non-motor symptoms. However, targeting TLR2 and TLR4 in the gut may exert multiple benefits, when administered alone or in combination with current PD therapeutics. The beneficial effects could include alleviating microbial dysbiosis and gut inflammation to improve gastrointestinal symptoms; improving metabolism of oral PD medications and their absorption at the gut barrier to increase medication efficacy and thus lessen the side effects of increasing dosage; and ameliorating systemic inflammation, which can all have downstream benefits on the CNS. Similarly, considering the hypothesised gut-brain spread of PD pathology in a subset of individuals, targeting TLR2/4 in the gut through lifestyle interventions, pre/probiotics or specific therapeutics could be very beneficial in early stages of the disease to alleviate α-synuclein aggregation and pathology. As such, TLR2 and TLR4 are potential targets for the development of novel therapies to modify the disease course in people at high risk of developing PD, those in prodromal stages, or those already diagnosed with the disorder. However, such therapeutic utility is currently limited, especially given the wide-spread and complex role of TLR signalling throughout the body and the heterogenous nature of PD. Consequently, further investigations into the contribution of TLR2/4-mediated gut dysfunction to PD pathogenesis and pathology are critically needed.

## Data Availability

Not applicable.

## Abbreviations

AKT: Protein kinase B
6-OHDA: 6-Hydroxydopamine
CD14: Cluster of differentiation 14
CNS: Central nervous system
DAMP: Damage-associated molecular pattern
ENS: Enteric nervous system
HMGB1: High-mobility group box protein 1
IBD: Inflammatory bowel disease
IEC: Intestinal epithelial cell
IL-10: Interleukin 10
LBP: Lipopolysaccharide binding protein
LPS: Lipopolysaccharides
MD-2: Myeloid differentiation factor-2 protein
MPTP: 1-Methyl-4-phenyl-1,2,3,6-tetrahydropyridine
MyD88: Myeloid differentiation primary response protein 88
NF-κB: Nuclear factor kappa-light-chain-enhancer of activated B cells
PAMP: Pathogen-associated molecular pattern
PD: Parkinson’s disease
PI3K: Phosphatidylinositol 3-kinase
PPARγ: Peroxisome proliferator-activated receptor-γ
TLR2: Toll-like receptor 2
TLR4: Toll-like receptor 4
TNF-α: Tumour necrosis factor-α
